# Optimal Weights Mixed Filter for removing mixture of Gaussian and impulse noises

**DOI:** 10.1371/journal.pone.0179051

**Published:** 2017-07-10

**Authors:** Qiyu Jin, Ion Grama, Quansheng Liu

**Affiliations:** 1 School of mathematical science, Inner Mongolia University, No. 235 Daxue West Road, Hohhot, 010021, China; 2 UMR 6205, Laboratoire de Mathmatiques de Bretagne Atlantique, Université de Bretagne-Sud, Campus de Tohaninic, BP 573, 56017 Vannes, France; 3 School of Computer and Software, Nanjing University of Information Science and Technology, Nanjing 210044, China; Beijing University of Technology, CHINA

## Abstract

In this paper we consider the problem of restoration of a image contaminated by a mixture of Gaussian and impulse noises. We propose a new statistic called ROADGI which improves the well-known Rank-Ordered Absolute Differences (ROAD) statistic for detecting points contaminated with the impulse noise in this context. Combining ROADGI statistic with the method of weights optimization we obtain a new algorithm called Optimal Weights Mixed Filter (OWMF) to deal with the mixed noise. Our simulation results show that the proposed filter is effective for mixed noises, as well as for single impulse noise and for single Gaussian noise.

## 1 Introduction

Noise can be systematically introduced into digitized images during acquisition and transmission, which usually degrade the quality of digitized images. However, various image-related applications, such as aerospace, medical image analysis, object detection etc., generally require effective noise suppression to produce reliable results. The nature of the problem depends on the type of noise to the image. Generally, two noise models can adequately represent most noise added to images. Often in practice it is assumed that the noise has two components: an additive Gaussian noise and an impulse noise.

The additive Gaussian noise model is defined by

$$\begin{array}{c} Y(x)=f(x)+\epsilon (x),\;x\in I, \end{array}$$ (1)

where $I={\left\{\frac{1}{N},\frac{2}{N},\cdots ,\frac{N-1}{N},1\right\}}^{2},$ *N* ∈ **N**, *Y* is the observed image brightness, *f*: **I** ↦ [*c*, *d*] is an unknown target regression function, and *ϵ*(*x*), *x* ∈ **I**, are independent and identically distributed (i.i.d.) Gaussian random variables with mean 0 and standard deviation *σ* > 0. The Model (1) adds to each digitized image pixel a value from a zero-mean Gaussian distribution. Such noise is usually introduced during image acquisition. The zero-mean property of this Gaussian distribution makes it possible to remove the Gaussian noise by Non-Local weighted averaging. Important denoising methods for the Gaussian noise model have been well developed in recent years, see for example [1–15]

The random impulse noise model is defined by

$$\begin{array}{c} Y(x)=\{\begin{array}{cc} n(x), & \text{if}\;x\in B,\\ f(x), & \text{if}\;x\in I\backslash B, \end{array} \end{array}$$ (2)

where **B** is the set of pixels contaminated by impulse noise, P(B)=p is the impulse probability (the proportion of the occurrence of the impulse noise), *n*(*x*) are independent random variables uniformly distributed on some interval [*c*, *d*]. The impulse noise is characterized by replacing a pixel value with a random one. Such a noise can be introduced due to transmission errors, malfunctioning pixel elements in the camera sensors, faulty memory locations, and timing errors in analog-to-digital conversion. Recently, some important methods have been proposed to remove the impulse noise, see for example: [16–23].

However, the above mentioned methods are not effective when we apply them to remove a mixture of the Gaussian and impulse noises defined by

$$\begin{array}{c} Y(x)=\{\begin{array}{cc} n(x), & \text{if}\;x\in B,\\ f(x)+\epsilon (x), & \text{if}\;x\in I\backslash B. \end{array} \end{array}$$ (3)

The Gaussian noise removal methods cannot adequately remove impulse noise, for they interpret the impulse noise pixel as edges to be preserved; when impulse removal methods are applied to an image corrupted with the Gaussian noise, such filters, in practice, leave grainy, visually disappointing results. Garnett et al. [24] introduced a new local image statistic called Rank Ordered Absolute Difference (ROAD) to identify the impulse noisy pixels and incorporated it into a filter designed to remove the additive Gaussian noise. As a result they have obtained a trilateral filter capable to remove mixed Gaussian and impulse noise. This method also performs well for removing the single impulse noise. A variant of the ROAD statistic called ROLD was introduced in Dong et al. [22] which amplifies the differences between noisy and noise-free pixels, so that the noise detection becomes more accurate. An impulse detector and a filter which efficiently removes impulse/Gaussian mixed noise has been proposed in Xiong and Yin [25]. Lien et al [26] employed a decision-tree-based impulse noise detector and an edge-preserving filter to reconstruct the intensity values of noisy pixels, whose hardware cost was low. For other developpements in this direction we refer to [27–30]. Recently Delon and Desolneux [31, 32] and Hu et al. [33] introduced patch-based approaches to deal with the impulse noise and the mixture of Gaussian and impulse noises.

In this paper, we propose a new patch-based filter that we call Optimal Weights Mixed Filter (OWMF), by improving the ROAD statistic of [24] and combining it with the Optimal Weights Filter in [11]. We introduce a new statistic called ROADGI (Rank-Ordered Absolute Differences for mixture of Gaussian and Impulse noises) which detects more effectively the impulse noise when it is mixed with Gaussian noise. The ROADGI statistic will give a weight for all pixels in the image, which take value in the interval (0, 1]. The weight will get low value (near to 0) when a pixel is contaminated by impulse noise; otherwise, it will carry a high value (near to 1). The ROADGI statistic is then combined with the Optimal Weights Filter (OWF) to deal with the mixed noise, by assigning nearly 0 weights for impulse noise points. The simulation results show that the proposed filter can effectively remove the mixture of impulse noise and the Gaussian noise. Moreover, when applied to either the single impulse noise or the single Gaussian noise it performs as good as the best filters specialized to single noises.

Let us point out the differences with the patch-based approaches in Delon and Desolneux [31, 32] and Hu et al. [33] which are all adapted for the mixed noise. The method in [31, 32] consists in finding the *n* most similar patches according to a suitably chosen distance between patches, with which one then constructs a maximum likelihood estimator. The filter in [33] is an extension of the Non-Local Means filter to the case of mixed noise, with weights depending on the ROAD statistic. In the present paper we use the optimal weights approach from [11, 34] and an improved version of ROAD statistic to appropriately measure the impact of the impulse noise pixels.

The rest of the paper is organized as follows. In Section 2 after a short recall of the Optimal Weights Filter and a brief presentation of the Trilateral Filter whose main ideas will be used in the definition of our new filter, we introduce our filter. In section 3, we provide visual examples and numerical results that demonstrate our method’s soundness. Section 4 is a brief conclusion.

## 2 Algorithms

### 2.1 Optimal Weights Filter

For any pixel *x*_0_ ∈ **I** and a given *h* > 0, the square window of pixels

$$\begin{array}{c} U_{x_{0},h}=\{x\in I\;:\|x-x_{0}{\|}_{\infty }\leq h\} \end{array}$$ (4)

will be called *search window* at *x*_0_, where *h* is a positive integer. The size of the square search window **U**_*x*_0_,*h*_ is the positive integer number *M* = (2*h* + 1)^2^ = card **U**_*x*_0_,*h*_. For any pixel *x* ∈ **U**_*x*_0_,*h*_ and a given integer *η* > 0 a second square window of pixels **V**_*x*,*η*_ = **U**_*x*,*η*_ will be called for short a *patch window* at *x* in order to be distinguished from the search window **U**_*x*_0_,*h*_. The size of the patch window **V**_*x*,*η*_ is the positive integer *m* = (2*η* + 1)^2^ = card **V**_*x*_0_,*η*_. The vector **Y**_*x*,*η*_ = (*Y* (*y*))_*y*∈**V**_*x*,*η*__ formed by the values of the observed noisy image *Y* at pixels in the patch **V**_*x*,*η*_ will be called simply *data patch* at *x* ∈ **U**_*x*_0_,*h*_. For any *x*_0_ ∈ **I** and any *x* ∈ **U**_*x*_0_,*h*_, a distance between the data patches **Y**_*x*,*η*_ = (*Y* (*y*))_*y*∈**V**_*x*,*η*__ and **Y**_*x*_0_,*η*_ = (*Y* (*y*))_*y*∈**V**_*x*_0_,*η*__ is defined by

$$\begin{array}{c} d^{2}\;(Y_{x,\eta },Y_{x_{0},\eta })=\frac{1}{m}\;{\left\|Y_{x,\eta }-Y_{x_{0},\eta }\right\|}_{2}^{2}, \end{array}$$ (5)

where

$$\begin{array}{c} {\left\|Y_{x,\eta }-Y_{x_{0},\eta }\right\|}_{2}^{2}=\sum_{y\in V_{x_{0},\eta }}{\left(Y\left(T_{x}y\right)-Y\;\left(y\right)\right)}^{2} \end{array}$$

and *T*_*x*_ is the translation mapping: *T*_*x*_ *y* = *x* + (*y* − *x*_0_). If we use the approximation

$${\left(f\left(T_{x}y\right)-f\left(y\right)\right)}^{2}\approx {\left(f\left(x\right)-f\left(x_{0}\right)\right)}^{2}=\rho_{f,x_{0}}^{2}\left(x\right)$$

and the law of large numbers, it seems reasonable that

$$\begin{array}{c} \rho_{f,x_{0}}^{2}\left(x\right)\approx d^{2}\left(Y_{x,\eta }-Y_{x_{0},\eta }\right)-2\sigma^{2}. \end{array}$$ (6)

For our filter, however, ne need an estimate for *ρ*_*f*,*x*_0__(*x*) without the square. As shown in [11], in practice, good denoising results are obtained by using the following approximation

$$\begin{array}{c} \rho_{f,x_{0}}\left(x\right)\approx {\hat{\rho }}_{x_{0}}\left(x\right)={\left(d\left(Y_{x,\eta }-Y_{x_{0},\eta }\right)-\sqrt{2}\sigma \right)}^{+} \end{array}$$ (7)

rather than extracting the root in Eq (6). The fact that ${\hat{\rho }}_{x_{0}}\left(x\right)$ is a reasonable estimator of *ρ*_*f*,*x*_0__ was justified by the convergence results in [11] (cf. Theorems 3 and 4 of [11]). The Optimal Weights Filter is defined by

$$\begin{array}{c} \text{OWF}\left(f\right)\left(x_{0}\right)=\frac{\sum_{x\in U_{x_{0},h}}\kappa_{\text{tr}}\left(\frac{{\hat{\rho }}_{x_{0}}(x)}{\hat{a}}\right)Y\;\left(x\right)}{\sum_{y\in U_{x_{0},h}}\kappa_{\text{tr}}\left(\frac{{\hat{\rho }}_{x_{0}}(x)}{\hat{a}}\right)}, \end{array}$$ (8)

where *κ*_tr_ is the usual triangular kernel:

$$\begin{array}{c} \kappa_{\text{tr}}(t)={\left(1-|t|\right)}^{+},\;t\in R^{1}. \end{array}$$ (9)

The bandwidth $\hat{a}>0$ is the solution of

$$\sum_{x\in U_{x_{0},h}}{\hat{\rho }}_{x_{0}}\left(x\right){\left(\hat{a}-{\hat{\rho }}_{x_{0}}\left(x\right)\right)}^{+}=\sigma^{2},$$

and can be calculated as follows. We sort the set $\left\{{\hat{\rho }}_{x_{0}}\left(x\right):x\in U_{x_{0},h}\right\}$ in the ascending order $0={\hat{\rho }}_{x_{0}}\left(x_{1}\right)\leq {\hat{\rho }}_{x_{0}}\left(x_{2}\right)\leq \cdots \leq {\hat{\rho }}_{x_{0}}\left(x_{M}\right)<{\hat{\rho }}_{x_{0}}\left(x_{M+1}\right)=+\infty$, where *M* = card **U**_*x*_0_,*h*_. Let

$$\begin{array}{c} a_{k}=\frac{\sigma^{2}+\sum_{i=1}^{k}{\hat{\rho }}_{x_{0}}{\left(x_{i}\right)}^{2}}{\sum_{i=1}^{k}{\hat{\rho }}_{x_{0}}\left(x_{i}\right)},\;1\leq k\leq M, \end{array}$$ (10)

and

$$\begin{array}{ccc} k^{*} & = & \max\{1\leq k\leq M\;:\;a_{k}\geq {\hat{\rho }}_{x_{0}}\left(x_{k}\right)\}\\ & = & \min\{1\leq k\leq M\;:\;a_{k}<{\hat{\rho }}_{x_{0}}\left(x_{k}\right)\}-1, \end{array}$$ (11)

with the convention that *a*_*k*_ = ∞ if ${\hat{\rho }}_{x_{0}}\left(x_{k}\right)=0$ and that min⌀=M+1. The solution can be expressed as $\hat{a}=a_{k^{*}}$; moreover, k* is the unique integer *k* ∈ {1, ⋯, *M*} such that $a_{k}\geq {\hat{\rho }}_{x_{0}}\left(x_{k}\right)$ and $a_{k+1}<{\hat{\rho }}_{x_{0}}\left(x_{k+1}\right)$ if *k* < *M*.

The proof of Remark 2.1 can be found in [11].

### 2.2 ROAD statistic and trilateral filter

In [24], Garnett et al introduced the Rank-Ordered Absolute Differences (ROAD) statistic to detect points contaminated by impulse noise. For any pixel *x*_0_ ∈ **I** and a given *d* > 0, we define the square window of pixels

$$\Omega_{x_{0},d}^{0}=\{x\;:\;0<N\|x-x_{0}{\|}_{\infty }\leq d\},$$

where *d* is a positive integer. The square window will be called deleted neighborhood at *x*_0_. The ROAD statistic is defined by

$$\begin{array}{c} ROAD\left(x_{0}\right)=\sum_{i=1}^{K}r_{i}\left(x_{0}\right),\;x_{0}\in I, \end{array}$$ (12)

where *r*_*i*_(*x*_0_) is the *i*-th smallest term in the set $\left\{|Y\left(x\right)-Y\left(x_{0}\right)|:x\in \Omega_{x_{0},d}^{0}\right\}$ and $2\leq K<\text{card}\;\Omega_{x_{0},d}^{0}$. In [24] it is advised to use *d* = 1 and *K* = 4. Note that if *x*_0_ is an impulse noisy point, the value of *ROAD*(*x*_0_) is large; otherwise it is small.

Following [24] and [28] the authors define the “joint impulsivity” *J*_*I*_ (*x*_0_, *x*) between *x*_0_ and *x* as:

$$\begin{array}{c} J_{I}\;(x_{0},x)=\exp(-\frac{{\left(ROAD\left(x_{0}\right)+ROAD\left(x\right)\right)}^{2}}{2{\left(2\sigma_{J}\right)}^{2}}), \end{array}$$ (13)

where the function *J*_*I*_ (*x*_0_, *x*) assumes values in [0, 1] and the parameter *σ*_*J*_ controls the shape of the function *J*_*I*_ (*x*_0_, *x*). If *x*_0_ or *x* is an impulse noisy point, then the value of *ROAD*(*x*_0_) or *ROAD*(*x*) is large and *J*_*I*_ (*x*_0_, *x*) ≈ 0; otherwise, the value of *ROAD*(*x*_0_) and *ROAD*(*x*) are small and *J*_*I*_ (*x*_0_, *x*) ≈ 1. The trilateral filter (cf. [24]) is given by

$$\text{TriF}\left(v\right)\left(x_{0}\right)=\frac{\sum_{x\in U_{x_{0},h}}w\left(x\right)Y\;\left(x\right)}{\sum_{x\in U_{x_{0},h}}w\left(x\right)},$$

where

$$\begin{array}{c} w\left(x\right)=w_{S}\left(x\right)w_{R}{\left(x\right)}^{J_{I}\;\left(x_{0},x\right)}w_{I}{\left(x\right)}^{1-J_{I}\;\left(x_{0},x\right)}, \end{array}$$

$$\begin{array}{c} w_{S}\left(x\right)=e^{-\frac{|x-x_{0}{|}^{2}}{2\sigma_{S}^{2}}}, \end{array}$$

$$\begin{array}{c} w_{R}\left(x\right)=e^{-\frac{{\left(Y\;\left(x\right)-Y\;\left(x_{0}\right)\right)}^{2}}{2\sigma_{R}^{2}}}, \end{array}$$

$$\begin{array}{c} w_{I}\left(x\right)=e^{-\frac{ROAD{\left(x\right)}^{2}}{2\sigma_{I}^{2}}}. \end{array}$$

This filter has been shown to be very efficient in removing a mixed noise composed of a Gaussian and random impulse noise.

### 2.3 Optimal Weights Mixed Filter

The ROAD statistic (cf. [24]) provides a effective measure to detection the pixel contaminated by impulse. In this paper, we take into account the character of Gaussian noise and modify the ROAD statistic to better adapt to the mixture of impulse and Gaussian noises. Instead of the ROAD statistic Eq (12) we propose to use the statistic

$$\begin{array}{c} ROADGI\left(x_{0}\right)={\left(\frac{1}{K}\;\sum_{i=1}^{K}\;r_{i}\left(x_{0}\right)-\sigma \right)}^{+},\;x_{0}\in I, \end{array}$$ (14)

where *σ* is the standard deviation of the added Gaussian noise, *r*_*i*_(*x*_0_) is the *i*-th smallest term in the set $\left\{|Y\left(x\right)-Y\left(x_{0}\right)|:x\in \Omega_{x_{0},d}^{0}\right\}$, and $2\leq K<\text{card}\;\Omega_{x_{0},d}^{0}$. An advantage of the ROADGI statistic, compared to the ROAD statistic, is that it is relatively stable with respect to size *d* of the detection window $\Omega_{x_{0},d}^{0}$, and takes into account the Gaussian noise level *σ*. Let

$$\begin{array}{c} J\left(x,H\right)=\exp(-\frac{ROADGI{\left(x\right)}^{2}}{H^{2}}), \end{array}$$ (15)

be a weight to estimate whether the point is impulse one, where the parameter *H* controls the shape of the function. In the case when the pixel *x* is an impulse point then *ROADGI*(*x*) is large and *J*(*x*, *H*) ≈ 0; otherwise *ROADGI*(*x*) ≈ 0 and *J*(*x*, *H*) ≈ 1.

Now, we modify the Optimal Weights Filter [11] in order to treat the mixture of impulse and Gaussian noises. Similar to Eq (5), we define the impulse detection distance by

$$d_{J,\kappa }\;(Y_{x,\eta },Y_{x_{0},\eta })=\frac{{\left\|(Y_{x,\eta }-Y_{x_{0},\eta })\right\|}_{J,\kappa }}{\sqrt{\sum_{y^{\prime }\in V_{x_{0},\eta }}\kappa \left(y^{\prime }\right)}},$$

where

$$\begin{array}{cc} & \|Y_{x,\eta }-Y_{x_{0},\eta }{\|}_{J,\kappa }^{2}\\ & =\sum_{y\in V_{x_{0},\eta }}\kappa \left(T_{x}y\right)J\left(T_{x}y,H_{1}\right)J\left(y,H_{1}\right){\left(Y\;\left(T_{x}y\right)-Y\;\left(y\right)\right)}^{2}, \end{array}$$

and *κ* are some weights defined on **V**_*x*_0_,*η*_. The corresponding estimate of brightness variation *ρ*_*f*,*x*_0__(*x*) is given by

$$\begin{array}{c} {\hat{\rho }}_{J,\kappa ,x_{0}}\left(x\right)={\left(d_{J,\kappa }\;(Y_{x,\eta },Y_{x_{0},\eta })-\sqrt{2}\sigma \right)}^{+}. \end{array}$$ (16)

The best denoising results are obtained when the smoothing kernel *κ* is defined by

$$\begin{array}{c} \kappa \;(y)=\sum_{k=\max(1,j)}^{\eta }\;\frac{1}{{\left(2k+1\right)}^{2}} \end{array}$$ (17)

if ‖*y* − *x*_0_‖_∞_ = *j* for some *j* ∈ {0, 1, ⋯, *η*} and *y* ∈ **U**_*x*_0_,*η*_. It is possible to use as *k* the Gaussian kernel, but the results are a bit less precise.

**Fig 1:**
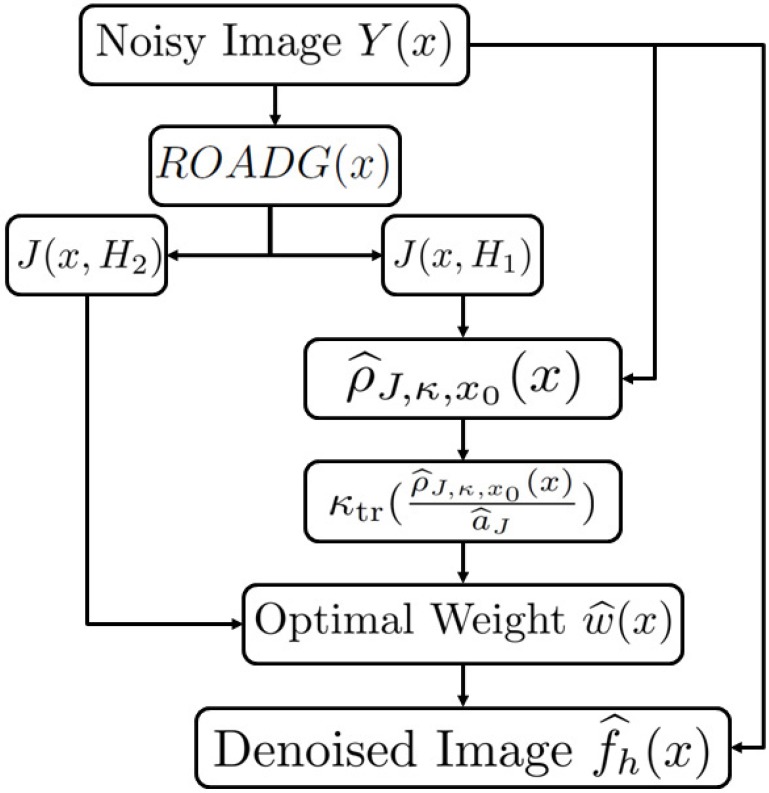
Flowchart of Optimal Weights Mixed Filter.

Now, we define a new filter, called *Optimal Weights Mixed Filter* (OWMF), by

$$\begin{array}{c} {\hat{f}}_{h}\left(x_{0}\right)=\frac{\sum_{x\in U_{x_{0},h}}\;J\left(x,H_{2}\right)\kappa_{\text{tr}}\left(\frac{{\hat{\rho }}_{J,\kappa ,x_{0}}(x)}{{\hat{a}}_{J}}\right)Y\;\left(x\right)}{\sum_{y\in U_{x_{0},h}}\;J\left(x,H_{2}\right)\kappa_{\text{tr}}\left(\frac{{\hat{\rho }}_{J,\kappa ,x_{0}}(x)}{{\hat{a}}_{J}}\right)}, \end{array}$$ (18)

where the bandwidth ${\hat{a}}_{J}>0$ can be calculated as in Remark 2.1 (with ${\hat{\rho }}_{x_{0}}\left(x\right)$ and $\hat{a}$ replaced by ${\hat{\rho }}_{J,\kappa ,x_{0}}\left(x\right)$ and ${\hat{a}}_{J}$ respectively) and *H*_2_ is a parameter. Notice that *H*_1_ and *H*_2_ may take different values. The flowchart and the pseudocode of algorithm of the OWMF are given by Fig 1 and Algorithm 1.

**Algorithm 1:** Optimal Weights Mixed Filter

 **Input:** Noisy image *Y*; The set of parameters {*d*, *K*, *M*, *m*, *H*_1_, *H*_2_}

 **Output:** Denoised image ${\hat{f}}_{h}$

1 **forall** *x* ∈ **I** **do**

2  compute $ROADGI\left(x\right)={\left(\frac{1}{K}\;\sum_{i=1}^{K}\;r_{i}\left(x\right)-\sigma \right)}^{+}$

3  compute $J\left(x,H_{1}\right)=\exp(-\frac{ROADGI{\left(x\right)}^{2}}{H_{1}^{2}})$

4  compute $J\left(x,H_{2}\right)=\exp(-\frac{ROADGI{\left(x\right)}^{2}}{H_{2}^{2}})$

5 **end**

6 **for** *each* *x*_0_ ∈ **I** **do**

7  give an initial value of $\hat{a}:\;\hat{a}=1$ (it can be an arbitrary positive number)

8  compute $\left\{{\hat{\rho }}_{J,\kappa ,x_{0}}\left(x\right)\;:\;x\in U_{x_{0},h}\right\}$ by Eq (16)

9  reorder $\left\{{\hat{\rho }}_{J,\kappa ,x_{0}}\left(x\right)\;:\;x\in U_{x_{0},h}\right\}$ as increasing sequence, say ${\hat{\rho }}_{J,\kappa ,x_{0}}\left(x_{1}\right)\leq {\hat{\rho }}_{J,\kappa ,x_{0}}\left(x_{2}\right)\leq \cdots \leq {\hat{\rho }}_{J,\kappa ,x_{0}}\left(x_{M}\right)$

10  **for** *k* = 1 *to* *M* **do**

11   **if** $\frac{\sigma^{2}+\sum_{i=1}^{k}\;{\hat{\rho }}_{r,\kappa ,x_{0}}^{2}\left(x_{i}\right)}{\sum_{i=1}^{k}\;{\hat{\rho }}_{r,\kappa ,x_{0}}\left(x_{i}\right)}\geq {\hat{\rho }}_{r,\kappa ,x_{0}}\left(x_{k}\right)$ **then** computer $\hat{a}=\frac{\sigma^{2}+\sum_{i=1}^{k}\;{\hat{\rho }}_{r,\kappa ,x_{0}}^{2}\left(x_{i}\right)}{\sum_{i=1}^{k}\;{\hat{\rho }}_{r,\kappa ,x_{0}}\left(x_{i}\right)}$;

12   **else** quit loop;

13  **end**

14  **forall** *x* ∈ **U**_*x*_0_,*h*_ **do**

15   $\hat{w}\left(x_{i}\right)=\frac{J\left(x,H_{2}\right)\kappa_{\text{tr}}\;(\frac{{\hat{\rho }}_{x_{0}}\left(x_{i}\right)}{\hat{a}})}{\sum_{x_{i}\in U_{x_{0},h}}\;J\left(x,H_{2}\right)\kappa_{\text{tr}}\;(\frac{{\hat{\rho }}_{x_{0}}\left(x_{i}\right)}{\hat{a}})}$

16  **end**

17  compute ${\hat{f}}_{h}\left(x_{0}\right):\;{\hat{f}}_{h}\left(x_{0}\right)=\sum_{x_{i}\in U_{x_{0},h}}\hat{w}\left(x_{i}\right)Y\;\left(x_{i}\right)$

18 **end**

19 To avoid the undesirable border effects, in our simulations we mirror the image outside the image limits symmetrically with respect to the border. At the corners, the image is extended symmetrically with respect to the corner pixels.

To explain the new algorithm Eq (18), note that the function *J*(*x*, *H*_2_) acts as a filter of the points contaminated by the impulse noise. In fact, if *x* is an impulse noisy point, then *J*(*x*, *H*_2_) ≈ 0. When the impulse noisy points are filtered, the remaining part of the image is treated as a image distorted by solely the Gaussian noise. So, in the new filter, the basic idea is to apply the OWF [11] by giving nearly 0 weights to impulse noisy points.

## 3 Simulation and comparisons

The performance of a filter $\hat{f}$ is measured by the usual Peak Signal-to-Noise Ratio (PSNR) in decibels (db) defined by

$$\begin{array}{c} PSNR=10\log_{10}\frac{255^{2}}{MSE}, \end{array}$$

$$\begin{array}{c} MSE=\frac{1}{\text{card}\;I}\;\sum_{x\in I}{\left(f\left(x\right)-{\hat{f}}_{h}\left(x\right)\right)}^{2}, \end{array}$$

where *f* is the original image.

In the simulations, to avoid the undesirable border effects in our simulations, we mirror the image outside the image limits. In more detail, we extend the image outside the image limits symmetrically with respect to the border. At the corners, the image is extended symmetrically with respect to the corner pixels.

In our simulations the parameters are chosen as follows:

$$\begin{array}{c} d=2, \end{array}$$

$$\begin{array}{c} K=12, \end{array}$$

$$\begin{array}{c} M=13\times 13, \end{array}$$

$$\begin{array}{c} m=15\times 15, \end{array}$$

$$\begin{array}{c} H_{1}=5+\frac{30}{1+20p}+{\left(\sigma -10\right)}^{+}\left(0.5-p\right), \end{array}$$

$$\begin{array}{c} H_{2}=27-20p. \end{array}$$

**Fig 2:**
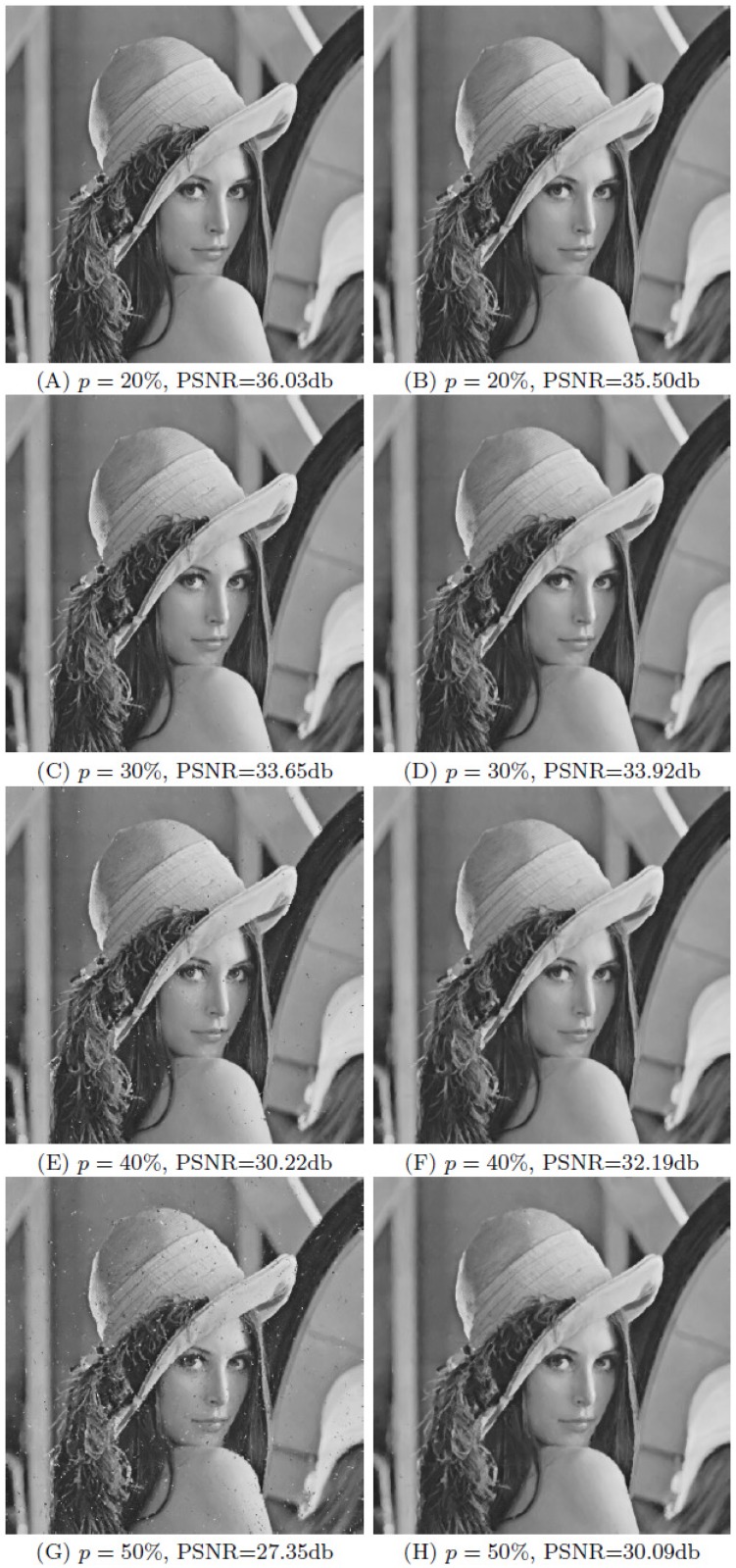
Restored images contaminated by pure impulse noise using our method (OWMF) with different sizes of the detection window $\Omega_{x_{0},d}^{0}$. The fist column corresponds to images restored with size 3 × 3 of the detection window. The second one is restored with size 5 × 5. The lines correspond to impulse noise proportions *p* = 20%, 30%, 40% and 50% respectively.

**Fig 3:**
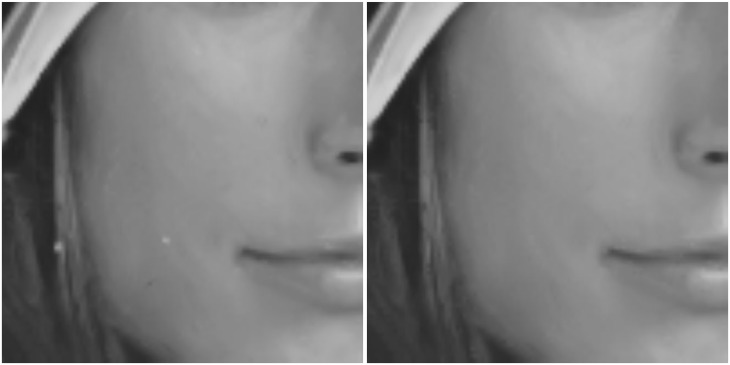
Details (of size 100 × 100) of the restored images contaminated by pure impulse noise using our method (OWMF) with different sizes of the detection window $\Omega_{x_{0},d}^{0}$. The first image is restored with the size 3 × 3 of detection window, the second one with the size 5 × 5. The original image has been contaminated by an impulse noise with *p* = 20%.

In [24] it is suggested to take *d* = 1 and *K* = 4. In [24], for low and moderate levels of noise (*p* < 25%), one iteration is sufficient and usually provides the best results; for high levels of noise (*p* > 25%), applying two to five iterations provides better results. Only one iteration is required in our simulations. If we choose *d* = 1 and *K* = 4, as recommended in [24], we found that a few spots of unremoved impulses often remain. This happens because impulses sometimes “clump” together, and the 3 × 3 detection window is too small to identify all the impulse noise points. Consequently, we select parameters *d* = 2 and *K* = 12 of detection windows for all levels of impulse noise. Fig 2 shows the comparison results between the restored images, with detection window 3 × 3 and with detection window 5 × 5, which have been added an impulse noise with *p* = 20%, 30%, 40%, and 50% respectively. When *p* = 30%, 40% and 50%, we can see clearly some impulse spots in the restored images with detection window 3 × 3, while the visual quality of the restored images with detection window 5 × 5 is very good, without impulse spots. In the case where *p* = 20%, impulse spots of the restored image with detection window 3 × 3 are not obvious, and the PSNR value is a little better than that with detection window 5 × 5, whereas Fig 3 shows that the first image has two clumpy impulse spots and the visual quality is not good enough. Consequently, we prefer detection window 5 × 5 for all levels impulse noise.

**Fig 4:**
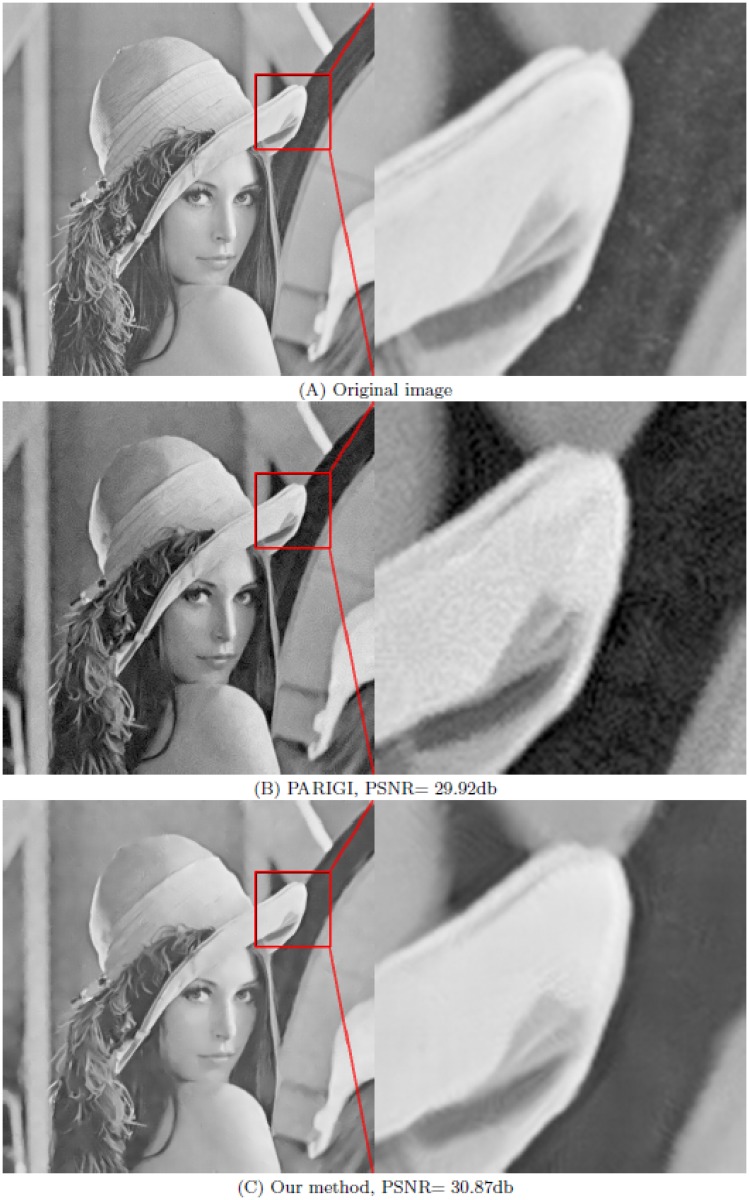
Comparison between PARIGI and our method (OWMF) for image “Lena” contaminated by Gaussian noise with *σ* = 20 and impulse noise with *p* = 20%. (A) the original image and its part; (B) the image restored by PARIGI and its part; (C) denoised image by our method and its part.

**Fig 5:**
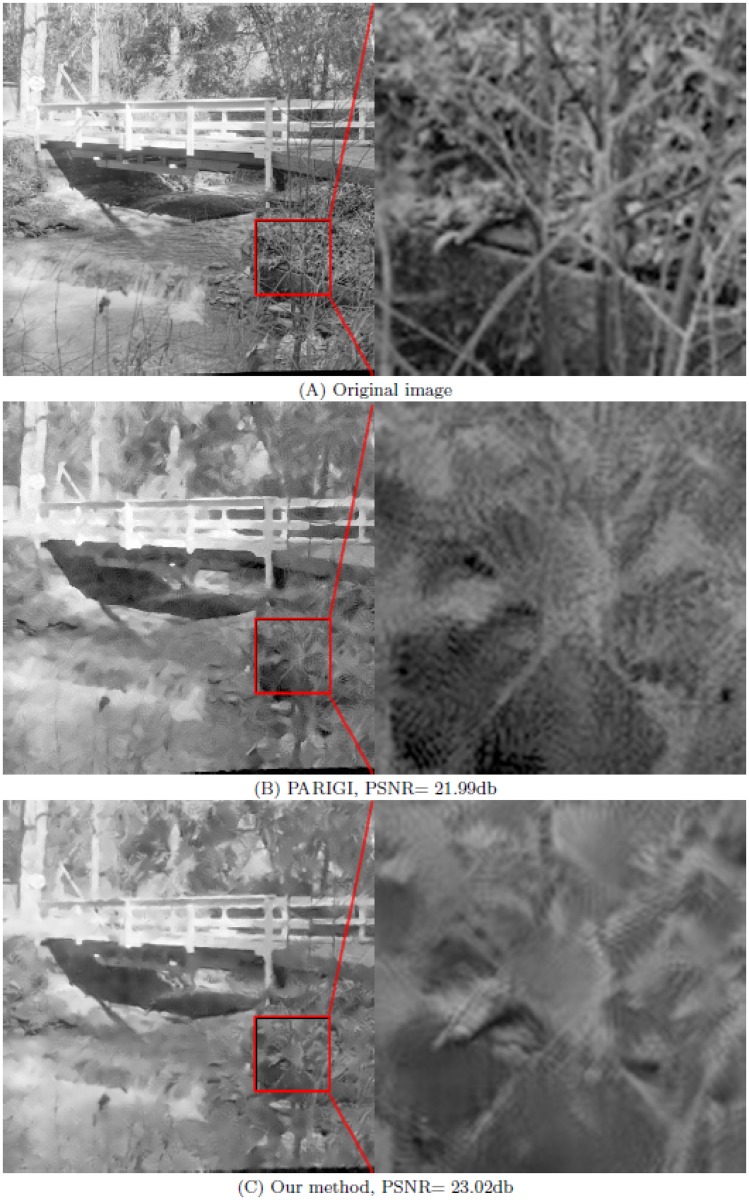
Comparison between PARIGI and our method (OWMF) for the image “Bridge” contaminated by Gaussian noise with *σ* = 30 and impulse noise with *p* = 30%. (A) the original image and its part; (B) the image restored by PARIGI and its part; (C) denoised image by our method and its part.

The parameters *m* and *M* have been fixed to *m* = 25 × 25 and *M* = 13 × 13. Figs 4(C) and 5(C) show that the noise is reduced in a natural manner and significant geometric features, fine textures, and original contrasts are visually well recovered with no undesirable artifacts. To better appreciate the accuracy of the restoration process, we zoom a part of the picture.

For comparison, we show the images denoised by PARIGI (see the left of Figs 4(B) and 5(B)) and their zoomed parts (see the right of Figs 4(B) and 5(B)). We can see clearly that the images denoised by our method are better than those denoised by PARIGI, so our method provides a significant improvement. The overall visual impression and the numerical results are improved using our algorithm.

For comparison, we consider the following three cases: pure Gaussian noise, pure impulse noise and the mixture of Gaussian and impulse noises.

In the case of pure Gaussian white noise, we have done simulation on a commonly-used set of images (“Lena”, “Barbara”, “Boat” and “House”) available at http://decsai.ugr.es/javier/denoise/test_images/ and the comparison with several filters is given in Table 1. The PSNR values show that our approach work as well as relatively sophisticated methods, like Hirakawa and Parks [35], Kervrann and Boulanger [36], Hammond and Simoncelli [7] and Aharon et al. [4], and is better than the filters proposed in Buades et al. [2], Katkovnik et al. [37], Foi et al. [38], Roth and Black [39], Hu et al. [33] and Delon et al. [32]. Exept [33] and [32], these methods can only deal with pure Gaussian noise, while our method can cope not only with the Gaussian noise, but also with the impulse noise and the mixture of Gaussian and pure impulse noises. The proposed approach gives a quality of denoising which is competitive with one of the state-of-the art methods, BM3D (see [5]).

**Table 1:** Comparison for removing Gaussian noise.

	Images	Lena	Barbara	Boat	House
	Sizes	512 × 512	512 × 512	512 × 512	256 × 256
*σ*	Method	PSNR	PSNR	PSNR	PSNR
15	Our method	33.75db	31.81db	31.02db	33.82db
	*M* = 13 × 13				
	*m* = 25 × 25				
	Buades et al. [2]	32.72db	31.67db	30.39db	33.82db
	Katkovnik et al. [37]	32.18db	29.20db	30.46db	32.62db
	Foi et al. [38]	32.72db	29.61db	30.93db	33.18db
	Roth and Black [39]	33.29db	30.16db	31.27db	33.55db
	Hirakawa and Parks [35]	33.97db	32.55db	31.59db	33.82db
	Kervrann and Boulanger [36]	33.70db	31.80db	31.44db	34.08db
	Jin et al. [11]	33.93db	32.31db	31.64db	34.09db
	Hammond and Simoncelli [7]	34.04db	32.25db	31.72db	33.72db
	Aharon et al. [4]	33.71db	32.41db	31.77db	34.25db
	Dabov et al. [5]	**34.27**db	**33.00**db	**32.14**db	**34.94**db
20	Our method	32.42db	30.40db	29.62db	32.71db
	*M* = 13 × 13				
	*m* = 27 × 27				
	Buades et al. [2]	31.51db	30.38db	29.32db	32.51db
	Katkovnik et al. [37]	30.74db	27.38db	29.03db	31.24db
	Foi et al. [38]	31.43db	27.90db	39.61db	31.84db
	Roth and Black [39]	31.89db	28.28db	29.86db	32.29db
	Hirakawa and Parks [35]	32.69db	31.06db	30.25db	32.58db
	Kervrann and Boulanger [36]	32.64db	30.37db	30.12db	32.90db
	Jin et al. [11]	32.68db	31.04db	30.30db	32.83db
	Hammond and Simoncelli [7]	32.81db	30.76db	30.41db	32.52db
	Aharon et al. [4]	32.39db	30.84db	30.39db	33.10db
	Dabov et al. [5]	**33.05**db	**31.78**db	**30.88**db	**33.77**db
	Hu et al. [33]	31.59db	- -.- -db	29.45db	- -.- -db
	Delon et al. [32]	27.51db	27.15db	26.55db	27.63db
25	Our method	31.40db	29.20db	28.56db	31.61db
	*M* = 13 × 13				
	*m* = 27 × 27				
	Buades et al. [2]	30.36db	29.19db	28.38db	31.16db
	Katkovnik et al. [37]	29.66db	26.05db	27.93db	30.12db
	Foi et al. [38]	30.43db	26.62db	28.60db	30.75db
	Roth and Black [39]	30.57db	26.84db	28.57db	31.05db
	Hirakawa and Parks [35]	31.69db	29.89db	29.21db	31.60db
	Kervrann and Boulanger [36]	31.73db	29.24db	29.20db	32.22db
	Jin et al. [11]	31.59db	29.92db	29.16db	31.95db
	Hammond and Simoncelli [7]	31.83db	29.58db	29.40db	31.54db
	Aharon et al. [4]	31.36db	29.58db	29.32db	32.07db
	Dabov et al. [5]	**32.08**db	**30.72**db	**29.91**db	**32.86**db

For the pure impulse noise, our method is also competitive. We choose a commonly used set of images “Baboon”, “Bridge”, “Lena” and “Pentagon”(where “Baboon”, “Bridge”, “Lena” and “Pentagon” available at http://www.math.cuhk.edu.hk/rchan/paper/dcx/), which is considered in Delon et al. [22]. Table 2 lists the restoration results using various known algorithms. It is clear that our method provides a significant improvement over Sun and Neuvo [40], Abreu et al. [17], Wang and Zhang [41], Chen et al. [42], Chen and Wu [18, 43], Crnojevic et al. [44], Wenbin [21], etc. Our approach works as well as Dong et al. [22], Yu et al. [23], Hu et al. [33] and Delon et al. [32]. It produces the best PSNR values in the cases of “Baboon” (40%) and “Pentagon” (40%), while Yu et al. [23] has the best results in the case of “Baboon” (20%) and “Bridge” (40%), and Dong et al. [22](ROLD-EPR) wins in the case of “Lena” (20% and 40%). Finally, in Table 3 we compare Garnett et al. [24], Hu et al. [33], Delon et al. [32] and our filter (OWMF) on the set of images “Lena”, “Bridge”, “Boat” and “Barbara”; from Table 3, it is clear that our method performs better. in most cases, especially when *σ* > 10.

**Table 2:** Comparison for removing impulse noise.

Images	Baboon		Bridge		Lena		Pentagon	
p%	20%	40%	20%	40%	20%	40%	20%	40%
Method	PSNR	PSNR	PSNR	PSNR	PSNR	PSNR	PSNR	PSNR
Our method	24.81db	**22.12**db	27.84db	24.91db	35.50db	32.19db	30.91db	**28.34**db
*M* = 13 × 13								
*m* = 25 × 25								
Sun and Neuvo [40]	23.67db	20.85db	26.26db	22.66db	32.93db	27.90db	29.34db	26.26db
Abreu et al. [17]	23.81db	21.49db	26.56db	23.80db	35.71db	29.85db	30.38db	27.27db
Wang and Zhang [41]	23.43db	21.07db	26.33db	22.75db	35.09db	28.92db	29.18db	26.19db
Chen et al. [42]	23.73db	21.38db	26.52db	22.89db	34.21db	28.30db	29.29db	26.29db
Chen and Wu [18]	24.02db	21.52db	27.27db	23.55db	35.44db	29.26db	30.34db	27.04db
Chen and Wu [43]	24.17db	21.58db	27.08db	23.23db	36.07db	28.79db	30.23db	26.84db
Crnojevic et al. [44]	23.78db	21.56db	26.90db	23.83db	36.50db	31.41db	30.11db	27.33db
Wenbin [21]	24.18db	21.41db	27.05db	23.88db	36.90db	30.25db	30.42db	26.93db
Garnett et al. [24]	24.18db	21.60db	27.60db	24.01db	36.70db	31.12db	30.33db	27.14db
Chan et al. [19]	23.97db	21.62db	27.31db	24.60db	36.57db	32.21db	30.03db	27.35db
Dong et al. [22]	24.49db	21.92db	27.86db	24.79db	**37.45**db	**32.76**db	30.73db	27.73db
Yu et al. [23]	**24.86**db	22.06db	28.06db	**24.97**db	36.18db	32.03db	- -.- -db	- -.- -db
Hu et al. [33]	- -.- -db	- -.- -db	**28.10**db	24.74db	35.90db	31.98db	- -.- -db	- -.- -db
Delon et al. [32]	24.46db	21.86db	26.53db	24.06db	36.62db	31.94db	**31.18**db	28.19db

**Table 3:** Comparison for removing mixed noise.

Gaussian Noise	Image	Method	*p* = 0.2	*p* = 0.3	*p* = 0.4	*p* = 0.5
sigma = 10	Lena	Garnett et al. [24]	31.48db	29.87db	28.57db	27.31db
		Hu et al. [33]	32.93db	31.30db	- -.- -db	- -.- -db
		Delon et al. [32]	33.16db	**32.93**db	**32.19**db	**30.37**db
		Our method	**33.18**db	32.05db	30.90db	29.52db
	Bridge	Garnett et al. [24]	25.82db	24.92db	23.79db	22.28db
		Hu et al. [33]	26.35db	35.00db	- -.- -db	- -.- -db
		Delon et al. [32]	25.81db	24.59db	23.67db	22.45db
		Our method	**26.42**db	**25.19**db	**24.08**db	**23.08**db
	Boat	Garnett et al. [24]	28.61db	27.54db	26.22db	24.74db
		Hu et al. [33]	**29.91**db	28.38db	- -.- -db	- -.- -db
		Delon et al. [32]	29.55db	**28.43**db	27.02db	25.46db
		Our method	29.57db	28.22db	2**7.05**db	**25.92**db
	Barbara	Garnett et al. [24]	24.82db	24.00db	23.08db	22.33db
		Hu et al. [33]	- -.- -db	- -.- -db	- -.- -db	- -.- -db
		Delon et al. [32]	**30.94**db	**30.02**db	**28.67**db	**26.49**db
		Our method	28.47db	26.46db	24.83db	23.62db
sigma = 20	Lena	Garnett et al. [24]	28.85db	28.02db	27.10db	25.68db
		Hu et al. [33]	30.47db	29.38db	- -.- -db	- -.- -db
		Delon et al. [32]	29.92db	29.31db	29.15db	**28.19**db
		Our method	**30.87**db	**30.09**db	**29.19**db	28.14db
	Bridge	Garnett et al. [24]	23.56db	23.01db	22.47db	21.72db
		Hu et al. [33]	24.53db	23.70db	- -.- -db	- -.- -db
		Delon et al. [32]	23.38db	23.14db	22.75db	21.82db
		Our method	**24.70**db	**23.97**db	**23.21**db	**22.45**db
	Boat	Garnett et al. [24]	26.18db	25.46db	24.75db	23.79db
		Hu et al. [33]	27.74db	26.66db	- -.- -db	- -.- -db
		Delon et al. [32]	26.61db	26.34db	25.64db	24.21db
		Our method	**27.79**db	**26.93**db	**25.97**db	**25.08**db
	Barbara	Garnett et al. [24]	23.35db	22.95db	22.53db	21.84db
		Hu et al. [33]	- -.- -db	- -.- -db	- -.- -db	- -.- -db
		Delon et al. [32]	**27.54**db	25.70db	**24.99**db	23.08db
		Our method	27.50db	**25.95**db	24.43db	**23.33**db
sigma = 30	Lena	Garnett et al. [24]	27.26db	26.57db	25.58db	23.99db
		Hu et al. [33]	28.67db	27.65db	- -.- -db	- -.- -db
		Delon et al. [32]	27.27db	26.72db	26.67db	26.32db
		Our method	**29.12**db	**28.49**db	**27.76**db	**26.75**db
	Bridge	Garnett et al. [24]	22.88db	22.42db	21.87db	20.98db
		Hu et al. [33]	23.35db	22.72db	- -.- -db	- -.- -db
		Delon et al. [32]	22.31db	21.99db	21.70db	21.22db
		Our method	**23.56**db	**23.02**db	**22.49**db	**21.86**db
	Boat	Garnett et al. [24]	25.11db	24.55db	23.80db	22.62db
		Hu et al. [33]	26.23db	25.48db	- -.- -db	- -.- -db
		Delon et al. [32]	24.45db	23.32db	22.85db	22.27db
		Our method	**26.41**db	**25.79**db	**25.08**db	**24.26**db
	Barbara	Garnett et al. [24]	22.82db	22.46db	21.94db	21.10db
		Hu et al. [33]	- -.- -db	- -.- -db	- -.- -db	- -.- -db
		Delon et al. [32]	25.03db	**24.96**db	**24.59**db	21.40db
		Our method	**25.98**db	24.81db	23.72db	**22.81**db

For a mixture of Gaussian and impulse noises simulation results show that the new proposed filter OWMF is competitive with PARIGI from [31, 32] and the filter in [33]. Table 3 shows that the results of denoising using our filter are generally better than those of PARIGI and [33] in the cases of “Lena”, “Bridge” and “Boat” when *σ* > 10. For *σ* = 10 our filter gives the results close to the best, which are sometimes the best. When considering the pure impulse noise, our method improves PARIGI and [33] in most cases; for pure Gaussian noise, our method is better than PARIGI and [33] with a larger margin.

## 4 Conclusion

A new image denoising filter to deal with the mixture of Gaussian and impulse noises, based on weights optimization and the modified Rank-Ordered Absolute Differences statistic, is proposed. The implementation of the filter is straightforward. Our work leads to the following conclusions.

1. The improved Rank-Ordered Absolute Differences statistic, used in the new filter, detects effectively the impulse noise in the case of mixture of Gaussian and impulse noises. This statistic is well adapted for use with the Weights Optimization Filter of [11].
2. It is shown by simulations that the proposed filter is very efficient for removing both a mixture of impulse and Gaussian noises, and the pure impulse or pure Gaussian noise.
3. Our numerical results demonstrate that the new filter is competitive with the known filters.
